# *Pyropia plicata* sp. nov. (Bangiales, Rhodophyta): naming a common intertidal alga from New Zealand

**DOI:** 10.3897/phytokeys.21.4614

**Published:** 2013-03-22

**Authors:** Wendy A. Nelson

**Affiliations:** 1 National Institute of Water and Atmospheric Research, Private Bag 14-901, Wellington 6241, New Zealand; 2 School of Biological Science, University of Auckland, PO Box 92-019, Auckland 1142, New Zealand

**Keywords:** Bangiales, New Zealand, *Porphyra, Pyropia columbina*, *Pyropia plicata* sp. nov.

## Abstract

A commonly found red alga of the upper intertidal zone of New Zealand rocky coasts is described for the first time as *Pyropia plicata* **sp. nov.** This species has been incorrectly known as *Porphyra columbina* Mont. (now *Pyropia columbina* (Mont.) W.A.Nelson) for many years. *Pyropia plicata* is widespread and common, and it is readily distinguished from other species of bladed Bangiales in New Zealand by its distinctive morphology, with pleated blades attached by a central rhizoidal holdfast.

## Introduction

For many years the most commonly found and widespread species of bladed Bangiales in New Zealand has been incorrectly known as *Porphyra columbina* Mont. Based on material collected from the New Zealand subantarctic Auckland Islands (Montagne 1842, 1845), *Porphyra columbina* was the first species in the order described from the New Zealand region. The name *Porphyra columbina* has been applied to specimens with widely ranging growth forms and colour states found in diverse habitats from subantarctic to warm temperate areas of New Zealand, Australia, and South America (e.g. Howe 1914, Taylor 1947, Levring 1953, 1955, 1960, Chapman 1969, Womersley and Conway 1975, Acleto and Endo 1977, Ricker 1987, Ramírez and Santelices 1991, Adams 1994, Womersley 1994). Although there have been significantly different interpretations of the species concept in New Zealand (e.g. Laing 1928, Levring 1955, Chapman 1969), the name *Porphyra columbina* in New Zealand has been generally applied to a common species with a very distinctive rosette-like morphology and deeply folded or pleated blades, found in the upper intertidal zone of mainland shores, as treated and illustrated in Nelson and Conroy (1989) and Adams (1994: p. 143). This species was assigned the code “ROS54” by Broom et al. (1999) and has been referred to by this code in a number of subsequent publications (e.g. Hemmingson and Nelson 2002, Jones et al. 2004, Nelson et al. 2006, Sutherland et al. 2011).

The combination of targeted collections of members of the Bangialesfrom throughout the New Zealand region, and analyses of sequence data coupled with morphological and anatomical investigations, has revealed many undescribed species around the archipelago (e.g. Broom et al. 1999, Nelson et al. 2001, Broom et al. 2004, Nelson and Broom 2005, Nelson et al. 2006). Recent collections of bladed Bangiales from subantarctic regions revealed at least four distinct species present on the Auckland Islands. With these data and specimens, Nelson and Broom (2010) were able to re-examine the original concept of *Porphyra columbina* and the subsequent interpretations and applications of this name. They concluded that *Porphyra columbina* is not conspecific with the mainland rosette-forming species referred to as ROS54, but rather it is primarily distributed in cold temperate areas of the southern hemisphere. They confirmed its presence on Auckland, Campbell, Antipodes, Chatham and Falkland Islands, and established that it is rarely present on mainland New Zealand (i.e. only one collection from the southern coast of the South Island from more than 700 samples of bladed Bangiales sequenced from the New Zealand region).

Although the monophyly of the Bangiales had been shown to be well supported by a number of studies (e.g. Oliveira and Bhattacharya 2000, Müller et al. 2001, Saunders and Hommersand 2004), Oliveira et al. (1995) demonstrated that neither of the two genera traditionally recognised in the order on the basis of gametophyte morphology (*Bangia* for filaments, *Porphyra* for foliose species) were monophyletic. A series of subsequent studies (e.g. Müller et al. 1998, Broom et al. 1999, 2004, Oliveira and Bhattacharya 2000, Lindstrom and Fredericq 2003, Nelson et al. 2006, Lindstrom 2008) provided further evidence of the diversity within the order and the need for segregate genera. Sutherland et al. (2011) revised the order Bangiales, recognising 15 genera of which eight are foliose. *Porphyra columbina* is now placed in the genus *Pyropia* (*Pyropia columbina* (Mont.) W.A.Nelson).

The rosette-forming species of *Pyropia*, previously referred to as ROS54, is formally described here.

## Materials and methods

This study is based on specimens of foliose Bangiales collected from throughout the New Zealand region, particularly from the North, South and Chatham Islands from 1987 to 2012, as part of diversity surveys. Voucher material is deposited in the herbarium of the National Museum of New Zealand Te Papa Tongarewa (WELT, Thiers 2012). Selected examples have been used for molecular sequence analyses (e.g. Broom et al. 1999, Nelson et al. 2006, Sutherland et al. 2011) as well as cell wall polysaccharide investigations (Hemmingson and Nelson 2002). Terminology for packets of reproductive cells follows Nelson et al. (1999).

## Taxonomy

### Pyropia plicata

W.A.Nelson sp. nov.

http://species-id.net/wiki/Pyropia_plicata

**Figure 1.**
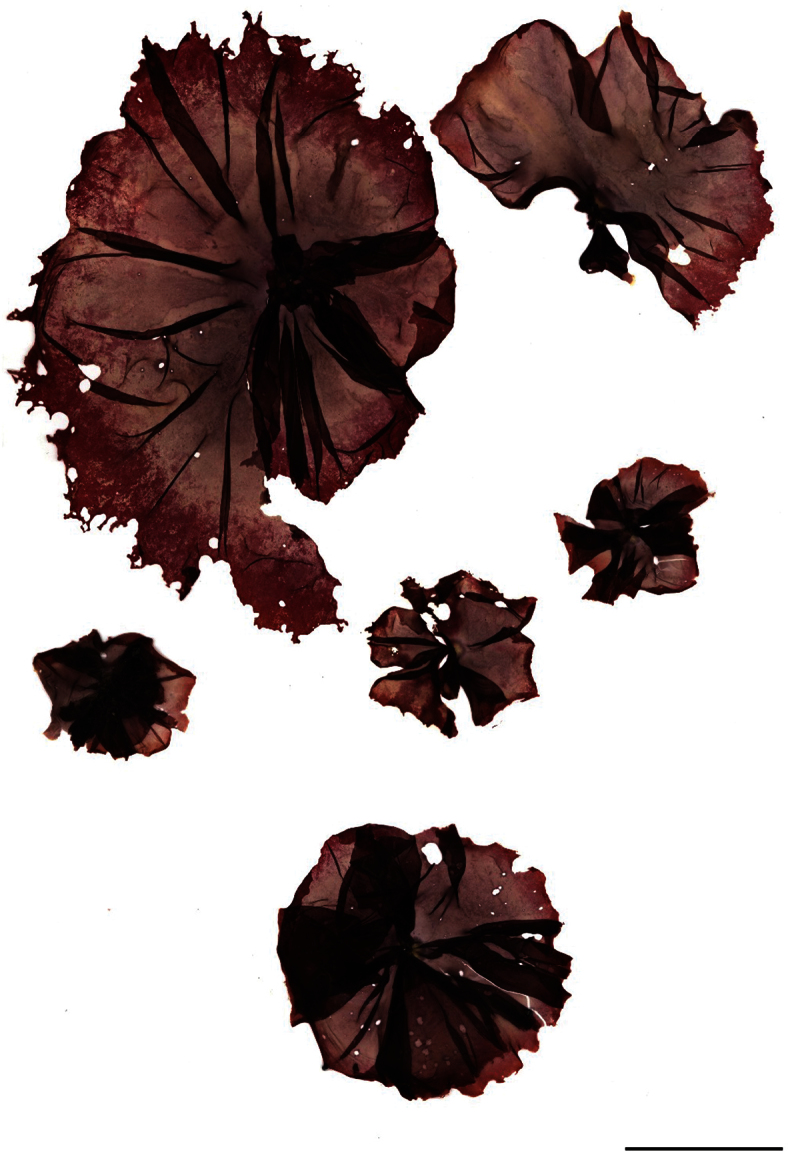
Holotype of *Pyropia plicata* sp. nov. (WELT A A032582). Scale bar = 5 cm.

Figures 1 [Fig F2] [Fig F3] –7

#### Diagnosis.

Blades circular to folded rosettes, strongly attached centrally by rhizoidal holdfast. Blades (1.5) 4–12 (42) cm in diameter. Colour purple to grey, bleaching to khaki-green on upper edges. Blades monostromatic, margin irregular bordered by pale cells. Monoecious, fertile regions marginal with intermixed sterile cells; zygotosporangia large, deep red to maroon, lozenge-shaped (a/4-8 × b/4-8 × c/4-8), spermatangia golden (a/2, b/2, c/8). Found in the upper to mid intertidal zone on open coasts.

#### Holotype.

WELT A032582 (Figure 1).

#### Type locality.

North Island, Wellington, Island Bay, W. Nelson, 22 Aug 1990.

#### Distribution.

New Zealand - North I., South I., Chatham Is.

#### Sequence data.

GenBank - nSSU – AF136426, *rbc*L – GU046410, voucher specimen = WELT A024408.

#### Etymology.

plicata – folded or pleated.

#### Description.

**Figure 2. F2:**
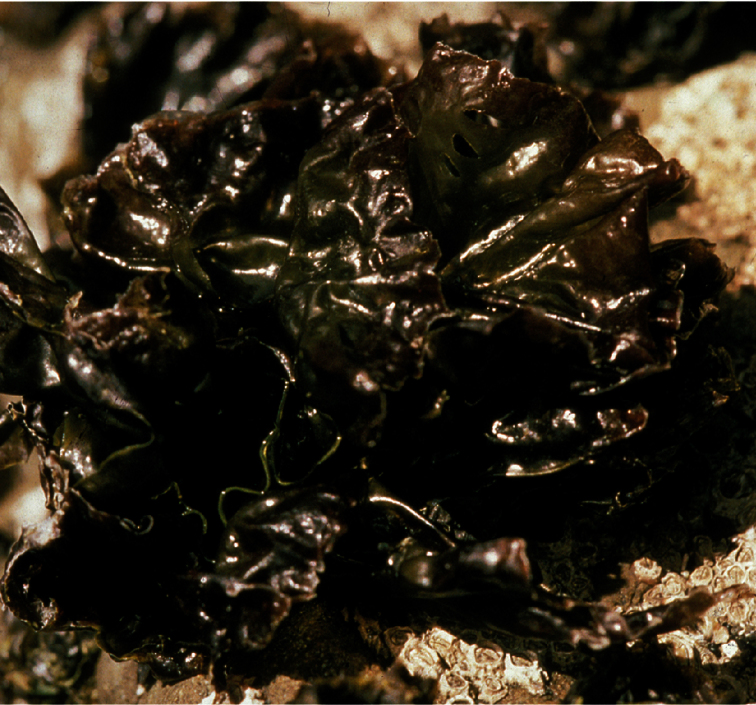
*Pyropia plicata* exposed at low tide on upper intertidal rocks (ca 5 cm high).

The blades of *Pyropia plicata* are deeply folded and when fully extended are seen to have a circular to oval shape. The blades are very variable in size, generally in the range of 4–12 cm in diameter although reproductively mature thalli have been found to range from 1.5 cm through to 42 cm in diameter. The thalli are attached to rock substrata by a centrally located holdfast, made up of rhizoids extending from cells in the lower (central) area of the blade. The thalli are robust and very strongly attached to rock substrata in the upper intertidal zone of rocky open coasts (Figure 2). Thalli are primarily purple to grey in colour, but they become bleached particularly in summer and autumn and become khaki to yellow-green particularly on the upper edges.

**Figure 3–4. F3:**
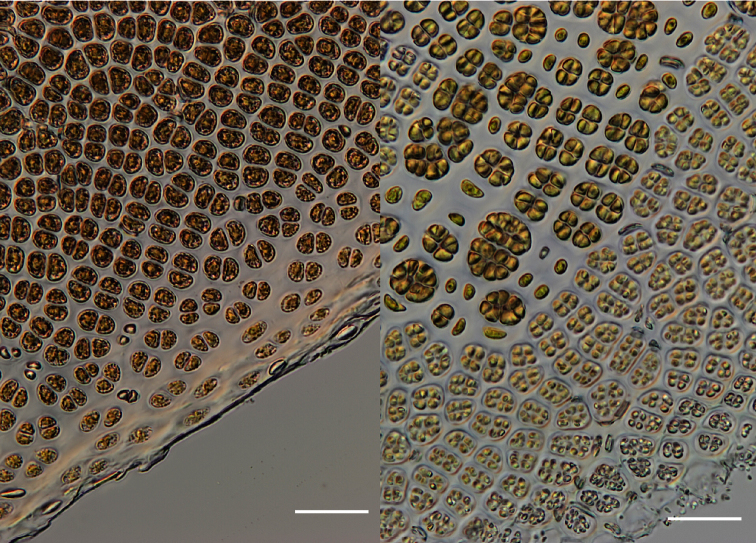
**3** (left): Vegetative region of the blade showing the margin with small pale cells. (WELT A032593) **4** (right): Fertile region of the blade with packets of developing zygotosporangia (larger, darker-coloured clusters), single sterile cells (between zygotosporangia) and packets of spermatangia (smaller, paler-coloured clusters) releasing at the blade margin. (WELT A032593). Scale bar = 50 µm.

Thalli are monostromatic and monoecious. Sterile regions of the blades are ca. 50-55 µm thick and the margin of the blade has a ragged or irregular appearance bordered by several layers of small pale cells (Figure 3). Fertile regions of the blade develop around the margins with sterile cells intermixed with patches of spermatangia and presumed zygotosporangia (Figure 4).

**Figure 5–7.**
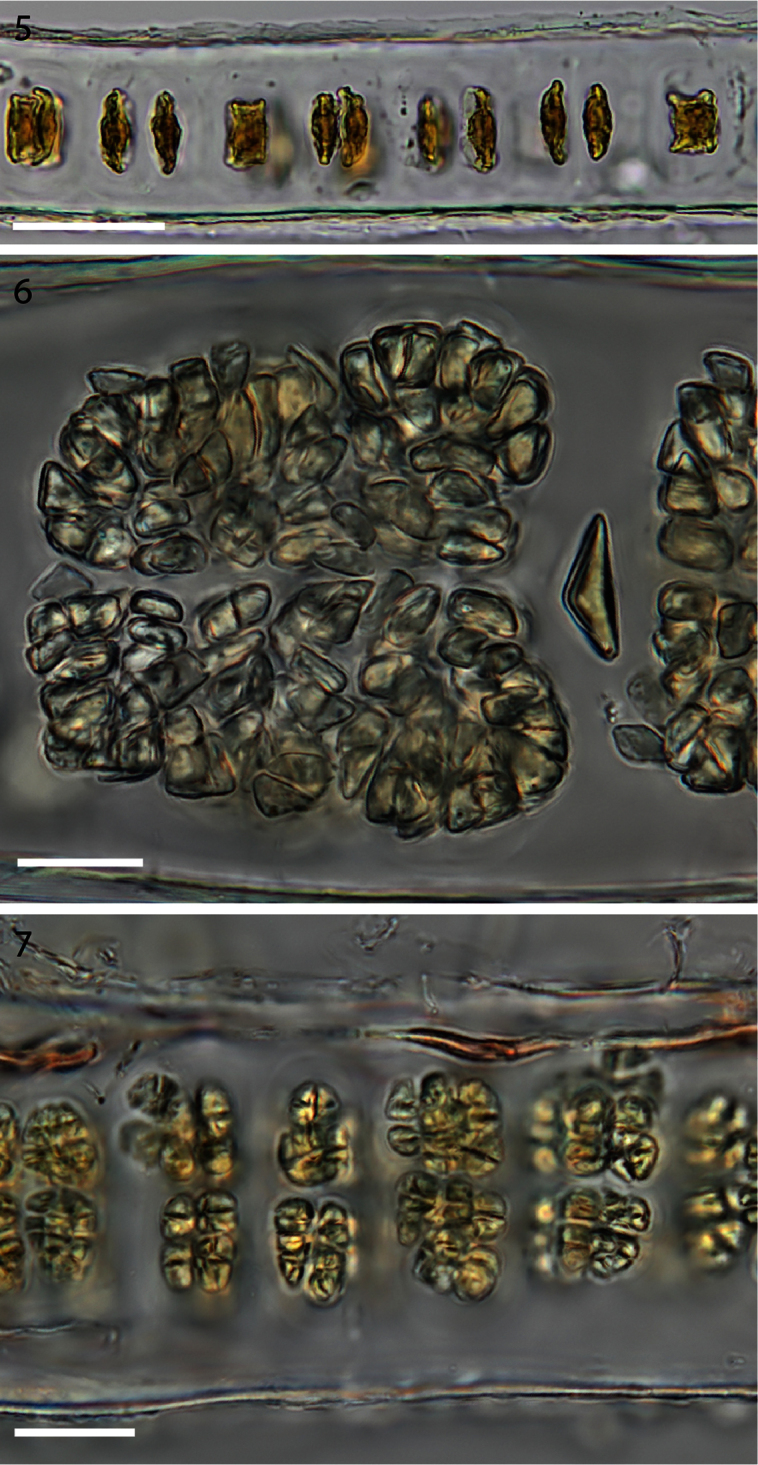
**5** Cross section of monostromatic blade showing square sterile cells and spindle-shaped developing carpogonia. (WELT A032593) **6** Cross section view of mature zygotosporangia. (WELT A032593) **7** Cross section view of mature spermatangia. (WELT A032593). Scale bar **5**: 50 µm, **6–7**: 20 µm.

In the early stages of development spindle-shaped carpogonia form trichogynes on both sides of the blade, in marked contrast to the box-like shape of the neighbouring sterile cells (Figure 5). Blades increase in thickness to ca. 85–110 µm in zygotosporangial regions (Figure 6) and ca. 60–70 µm in mature spermatangial regions (Figure 7). The zygotosporangia when mature are deep red and the packets vary in size, becoming lozenge shaped at maturity with divisions up to a/8, b/8, c/8 (Figure 6). The spermatangial patches become golden as they develop and when mature are divided into packets ca. a/2, b/2, c/8 (Figure 7). Spermatia and zygotospores are usually released before reaching the maximum division formulae.

Typically *Pyropia plicata* is found on the upper intertidal shores of open coasts on rocky substrata. It has not been found growing epiphytically and is uncommon in sheltered areas. The deep pleats and central attachment of *Pyropia plicata* enable the retention of moisture between the folds in the blade. This morphology would appear to be advantageous in the upper intertidal habitats where it is found, as this species can be out of water for periods of up to eight hours between tidal cycles. The outer part of a clump of *Pyropia plicata* may be dried with a cellophane-like appearance yet within the folds, parts of the blade remain wet.

*Pyropia plicata* shows no particular seasonal trends in its distribution, with reproductively mature specimens collected throughout the year. Collections of this species have been made from the northern tip of the North Island, through to areas on the south western and south eastern South Island, as well as on the Chatham Islands. It has not been found on the Three Kings Islands, Stewart Island, or any of the New Zealand subantarctic islands.

Distinctive features: *Pyropia plicata* can be distinguished from other New Zealand species of bladed Bangiales by a number of distinctive features. It is the only species of *Pyropia* present on mainland shores with a marked rosette-like growth form. Although the ribbon-like blades of *Pyropia cinnamomea* may become eroded with age, the basal position of the holdfast in this species differs from *Pyropia plicata*. In addition, these two species can be distinguished by colour, and also by the division formulae of zygotosporangia. On intertidal shores *Pyropia plicata* is characteristically found in the high intertidal but below the position occupied by *Clymene coleana* (W.A.Nelson) W.A.Nelson from which it can be easily distinguished. *Clymene coleana* has finely divided finger-like lobes rather than the continuous circular to oval deeply pleated blade of *Pyropia plicata*. Although both of these species have a predominantly grey colour in winter months, they bleach to different colours in bright light, with *Clymene coleana* becoming golden compared with the khaki colour of *Pyropia plicata*. In addition the zygotosporangia and spermatangia are arranged in separate areas of the blade in *Clymene coleana* rather than being intermixed in *Clymene plicata*.

#### Selected specimens examined:

**New Zealand. North Island.** **North Auckland:** Far North, east Tapotupotu Bay, 13 Nov 2001, R. Dunmore, WELT A030179 (34°26.1080'S, 172°43.0050'E); Muriwai Beach, Maori Bay (Maukatia), 04 Apr 2000, W. Nelson, T. Farr & G. Williams, WELT A024784 (36°50.30'S, 174°25.90'E). **Bay of Plenty:** Tauranga, Mount Maunganui main beach, 05 May 2000, G. Williams & T. Farr, WELT A024775 (37°38.00'S, 176°11.00'E); Maketu, Okurei Point East, 05 May 2000, T. Farr & G. Williams, WELT A024772 (37°44.95'S, 176°28.37'E). **Wellington:** Wellington City Harbour, Frank Kitts Lagoon reclamation, 12 Feb 2001, W. Nelson & T. Farr, WELT A030170 (41°17.20'S, 174°46.90'E); Lyall Bay, 5 Nov 2012, W. Nelson, WELT A032593 (41°21.0'S, 174°48.00'E; Southern Wairarapa, Ngawihi, 26 Oct 2000, W. Nelson, T. Farr & G. Williams, WELT A024816 (41°36.00'S, 175°14.00'E).

**Chatham Islands**. Reef at Owenga wharf, 10 Mar 2001, W. Nelson, J. Broom, W. Jones, T. Farr & M. Clayton, WELT A030169 (44°01.50'S, 176°22'W).

**South Island**. **Marlborough:** D’Urville Island, Bonne Point, 20 Sep 1999, W. Nelson & G. Williams, WELT A031087 (40°52.00'S, 173°55.00'E).

Kaikoura, Ocean View, 18 Oct 1997, W. Nelson, WELT A024408 (42°31'S, 173°30'E). **Westland:** West coast, Charleston, Constant Bay, 10 Mar 2000, W. Nelson & T. Farr, WELT A024727 (41°54.20'S, 171°26.00'E); Punakaiki, 12 Mar 2000, W. Nelson & T. Farr, WELT A024793 (42°06.70'S, 171°20.00'E). **Canterbury:** Banks Peninsula, Avon Heathcote estuary, 20 Mar 2000, J. Broom, WELT A023952 (43°33.00'S, 172°44.00'E); Christchurch, Sumner, Cave Rock, 20 Mar 2000, J. Broom, WELT A023953, also, 26 Jun 2005, J. Broom & S. Heesch, WELT A023946 (43°33.9370'S, 172°45.5190'E); Lyttelton Harbour, Corsair Bay, 17 Sep 2001, M. Parsons, W. Jones & K. Neill, WELT A030172 (43°37.00'S, 172°42.00'E). **Otago:** Purakanui, 19 Apr 2000, K. Neill, WELT A023949 (45°45.00'S, 170°38.00'E); Dunedin, Brighton, 22 Oct 1999, J. Broom & W. Nelson, WELT A023956 (45°57.05'S, 170°20.00'E). **Southland:** Catlins, Kaka Point, 28 Apr 2005, S. Heesch & J. Broom, WELT A031597 (46°23.010'S, 169°47.140'E); Fiordland, Edwardson Sound, Chalky Inlet, 23 Feb 2000, G. Williams, WELT A024786 (45°55.983'S, 166°38.067'E).

## Discussion

A major problem in Bangiales taxonomy has been the incorrect application of names, making studies of the ecology and comparative physiology of species exceedingly difficult. The need for molecular sequence data in Bangiales taxonomic studies has been emphasised by many authors over the past decade in order to clarify species concepts as well as the phylogenetic relationships amongst taxa (e.g. Lindstrom and Fredericq 2003, Nelson et al. 2006). Such data have led to the discovery of cryptic taxa amongst species with very similar morphologies (e.g. Brodie and Irvine 1997, Broom et al. 2002, 2004, Neefus et al. 2002, Lindstrom and Fredericq 2003, Brodie et al. 2007, Lindstrom 2008). Descriptions of foliose members of the Bangiales have traditionally emphasised features such as blade shape and size, colour, and texture, in addition to division formulae for spermatangia and phyllosporangia, number of cell layers, number of plastids (e.g. summarised in Lindstrom and Cole 1993). In addition to external morphology (including marginal structure) and reproductive features, Miyata and Kikuchi (1997) also found seasonality and habitat (whether species are epiphytic or epilithic) to be have value taxonomically when distinguishing species of bladed Bangiales in Japan.

As circumscribed by Sutherland et al. (2011), the genus *Pyropia* encompasses species displaying a wide range of morphological forms, a wide colour spectrum and at least four different types of arrangements of reproductive regions on sexual thalli. This genus is the most speciose of the Bangiales, and it also has the widest geographic distribution, with species occurring from tropical to cold temperate waters. *Pyropia plicata* has been recognised in the flora of mainland New Zealand for a long time, but has remained without a formal name as a result of confusion over the application of the name *Porphyra columbina*. This situation was able to be clarified only after material collected in the subantarctic islands became available for study (Nelson and Broom 2010). Although mature thalli of *Pyropia plicata* range widely in size and also in colour, the fundamental shape of the blade, and the arrangement of reproductive regions are consistent, and enable this species to be readily distinguished. Within the genus *Pyropia*, *Pyropia plicata* is grouped within a clade of at least 15 southern hemisphere species. The majority of these species are currently undescribed but the clade includes *Porphyra virididentata*, *Porphyra cinnomomea* and *Porphyra columbina* (Broom et al. 2010, Sutherland et al. 2011).

## Supplementary Material

XML Treatment for Pyropia plicata
